# A systematic review of diagnostic, prognostic, and risk blood and urine biomarkers of transplant-associated thrombotic microangiopathy

**DOI:** 10.3389/fimmu.2022.1064203

**Published:** 2023-02-02

**Authors:** Michelle L. Schoettler, Harshil Bhatt, Sumithira Vasu

**Affiliations:** ^1^ Children’s Healthcare of Atlanta/Aflac Cancer and Blood Disorders Center, Emory University, Atlanta, GA, United States; ^2^ Department of Pediatrics, Emory School of Medicine, Columbus, OH, United States; ^3^ Hematopoietic Cell Transplantation, Ohio State University, Columbus, OH, United States

**Keywords:** transplant associated thrombotic microangiopathy, biomarkers, complement activation, endothelial activation, sC5b-9 (serum complement membrane attack complex), NETs (neutrophil extracellular traps)

## Abstract

Transplant-associated thrombotic microangiopathy (TA-TMA) is an increasingly recognized complication of allogeneic and autologous hematopoietic cellular therapy (HCT), associated with significant morbidity and mortality. Although the central drivers of the disease are thought to be endothelial damage and complement activation, no specific diagnostic biomarkers have been identified. TA-TMA is typically diagnosed using criteria comprised of non-specific clinical and laboratory features. Some patients will have a self-remitting course, but more than half develop multi-organ dysfunction or die, making prognostic biomarkers critical. Prevention of TA-TMA, an approach central to other HCT complications such as graft-versus-host disease, is largely untested in part due to a lack of identified early high-risk biomarkers. We conducted a systematic review to summarize the diagnostic, early risk, and prognostic biomarkers of TA-TMA. We screened the titles and abstracts of 1524 citations. After screening out duplications, we read the abstracts of 979 papers and fully reviewed 132 full-text publications. Thirty-one publications fulfilled the inclusion criteria of more than five patients with TA-TMA and a reported measure of association with diagnosis, prognosis, or risk of later development of the disease. Fourteen studies (45%) were with adults, 12 (39%) were with children <18 years old, three included both children and adults, and two did not report age. There were 53 biomarker or biomarker signature entries, and a total of 27 unique biomarkers. Only four biomarkers reported sensitivity and specificity. The single biomarker with the most robust data was sC5b-9, which conferred diagnostic, prognostic, and risk implications. Studies of combinations of biomarkers were rare. No meta-analyses were performed because of significant heterogeneity between studies. The limitations of studies included small sample size, study designs with a high risk of bias (i.e., case–control), the timing of sample collection, and the selection of controls. Furthermore, only two (6%) studies included a training and validation cohort. Cut-off points are needed to stratify groups, as most biomarkers do not have normal values, or normal values cannot be assumed in the HCT setting. In the future, multi-institutional, collaborative efforts are needed to perform rigorously designed, prospective studies with serially enrolled patients, with samples collected at the time of TA-TMA diagnosis, careful selection of controls, and validation of selected biomarkers and cut-off points in a separate cohort.

## Introduction

Transplant-associated thrombotic microangiopathy (TA-TMA) is an increasingly recognized complication thought to complicate ~15%–30% of allogeneic hematopoietic cell transplantation (HCT) recipients. TA-TMA also occurs after autologous HCT, particularly in children with neuroblastoma (1–3). Multiple hits to the endothelium results in a pro-apoptotic, pro-inflammatory, and pro-thrombotic milieu and inappropriate complement activation resulting in further endothelial damage (4–7). Risk factors for TA-TMA include inherent features of underlying HCT diagnosis, age, sex, prior endothelial injury or complement genetic variations; donor-related complement genetic variations; transplant characteristics including HLA mismatch, myeloablative conditioning, radiation, and graft-versus-host disease (GVHD) prophylaxis; and transplant complications including GVHD and infection (3, 8–13).

TA-TMA shares the principal features of other TMAs—consumptive microangiopathic hemolytic anemia, platelet consumption, and organ dysfunction. Over 50% of patients with TA-TMA develop multiorgan dysfunction (MOD); once this develops mortality in untreated patients can exceed 80% (2). Clinical manifestations include diffuse alveolar hemorrhage, respiratory failure, pulmonary hypertension, kidney injury or failure, altered mental status, posterior reversible encephalopathy syndrome (PRES), seizures, bowel ischemia, abdominal pain, gastrointestinal bleeding, and pulmonary or cardiac effusions requiring drainage. Among patients who develop MOD mortality rates exceed 50% (14). Non-relapse mortality (NRM) remains significantly higher in TA-TMA patients even after adjusting for other concurrent co-morbidities (15, 16).

Early identification of TA-TMA is critical as there are treatments that impact survival and multiple ongoing clinical trials investigating new therapeutic agents (17–20). Although a renal biopsy is considered the gold standard for TA-TMA diagnosis, it is rarely obtained in the post-HCT setting and there are no identified specific diagnostic biomarkers of the disease. Thus, TA-TMA is primarily a clinical diagnosis. Several diagnostic criteria have been identified and all depend on a combination of non-specific features ( **Table 1** ) (2, 16, 21, 22). Despite expert consensus, there is not a uniformly accepted diagnostic criterion for TA-TMA (23). In addition to identifying patients with TA-TMA, prognostic markers are critical—the goal is to identify those patients at the highest risk for developing severe disease and who will most benefit from TA-TMA-directed therapy while sparing those who are most likely to have a self-remitting course from the side effects and the costs of the drug. Subclinical biomarkers predicting the risk of developing TA-TMA could facilitate strategies to pre-emptively treat or prevent TA-TMA, akin to GVHD and sinusoidal obstructive syndrome (SOS) (24, 25). Our objective for this systemic review was to identify existing diagnostic, prognostic, and risk biomarkers for TA-TMA and to evaluate the quality and the level of evidence around them.

**Table 1:** Clinical diagnostic criteria for TA-TMA.

Clinical marker	BMT-CTN	IWG	Cho et al.	City of Hope	Uderzo et al.	Jodele et al.
	All features present	All features present	All features present at least 2 time points	4 features, definite, 3 probable	All features present	≥4/7 criteria, at least 2 time points within 14 days
Anemia	–	+	+	–	+	+
Thrombocytopenia (1)	–	+	+	+	+	+
Negative Coombs test	+	–	+	–	+	–
Schistocytes	+	+	+	+	+	+
Decreased levels of haptoglobin	–	+	+	–	–	–
Elevated levels of serum LDH	+	+	+	+	+	+
Hypertension (2)	–	–	–	–	+	+
Proteinuria (3)	–	–	–	–	+	+
Renal or neurological dysfunction	+	–	–	+	+	+
Elevated levels of serum C5b-9	–	–	–	–	+	+

Bone Marrow Transplant and Clinical Trials Network (BMT-CTN), International Working Group (IWG), lactate dehydrogenase (LDH), high-power field (HPF), ^1^platelet count ≤ 50,000/μl or ≥ 50% reduction from baseline, ^2^blood pressure ≥99% for age (<18 years old); ≥140/90 mmHg (≥18 years old); resistant to ≥ 2 antihypertensive agents, ^3^random urine protein concentration ≥30 mg/dL.mg/dl.

## Methods

We conducted a systematic review of diagnostic, prognostic, and risk biomarkers in accordance with Preferred Reporting Items for Systematic Reviews and Meta-Analyses (PRISMA) standards. All studies of genetic variants and immunohistochemical staining of tissues were excluded, and no biomarkers from other fluid sources (i.e., bronchoscopy fluid or cerebrospinal fluid) were identified.

### Data sources and search strategy

A literature search was performed using Embase^®^ (Elsevier, Amsterdam, the Netherlands) and PubMed^®^ (National Library of Medicine, Bethesda, MD, USA) databases for relevant original studies from inception to 4 August 2022. There were no search filter, date, or language restrictions applied to the literature search to limit results. A broad search was conducted ( **Supplemental Table 1** ).

### Eligibility requirements

Studies were included if they were original human studies related to biomarkers in TA-TMA and included at least five patients with TA-TMA. Studies not related to TA-TMA, duplicate articles, review articles, animal studies, studies that included fewer than five patients with TA-TMA, studies with no biomarker data, abstracts that were published later, papers published only in conference proceedings, and studies of genetic variants in TA-TMA were excluded. There were no exclusion criteria regarding patient characteristics, TA-TMA diagnosis used, study design, or time.

### Data extraction and quality

Two reviewers (MS and HB) independently screened all potentially relevant titles and abstracts for eligibility. Data gathered included author and publication year, type of study design (cohort, case–control, hybrid, retrospective or prospective, blinded, or not blinded), biomarkers, number of TA-TMA cases, diagnostic criteria used for TA-TMA, controls, sample approach, age group of cohorts, and measure of association such as hazard ratios (HRs), odds ratios (ORs), or information on sensitivity/specificity for biomarkers.

### Assessment of quality and risk of bias of individual studies

A quality assessment of diagnostic accuracy studies (QUADAS)-2 assessment was performed independently by two authors (HB and MS). The QUADAS-2 is a tool that includes four domains designed to ascertain the risk of bias and standardize systematic reviews (26). Biomarker entries with available data on sensitivity/specificity and a post-HCT control group were included in the α-group, whereas β-group entries lacked either or both characteristics. For each group, biomarkers were categorized into four groups: markers of complement activation, endothelial activation, thrombosis, and miscellaneous. Finally, each of the biomarker groups were further divided into diagnostic, risk, and prognostic categories based on the data analysis.

### Biomarker definitions

Biomarkers were defined as per the Federal Drug Association and National Institutes of Health joint leadership guidelines. Diagnostic biomarkers detect or confirm a diagnosis. Risk biomarkers detect the potential for disease in patients without a clinically evident disease. Prognostic biomarkers determine the anticipated disease course in patients with clinically evident disease (27).

### Biomarker entry

Each biomarker or biomarker combination with either diagnostic, prognostic, or risk value was defined as a biomarker entry. Some manuscripts had multiple biomarker entries, resulting in several biomarker entries per paper. For example, if a single biomarker was tested in three ways—(1) predicting the development of later TA-TMA, (2) diagnostic ability, and (3) association of later non-relapsed related mortality (prognosis)—this resulted in three biomarker entries.

## Results

**Figure 1:**
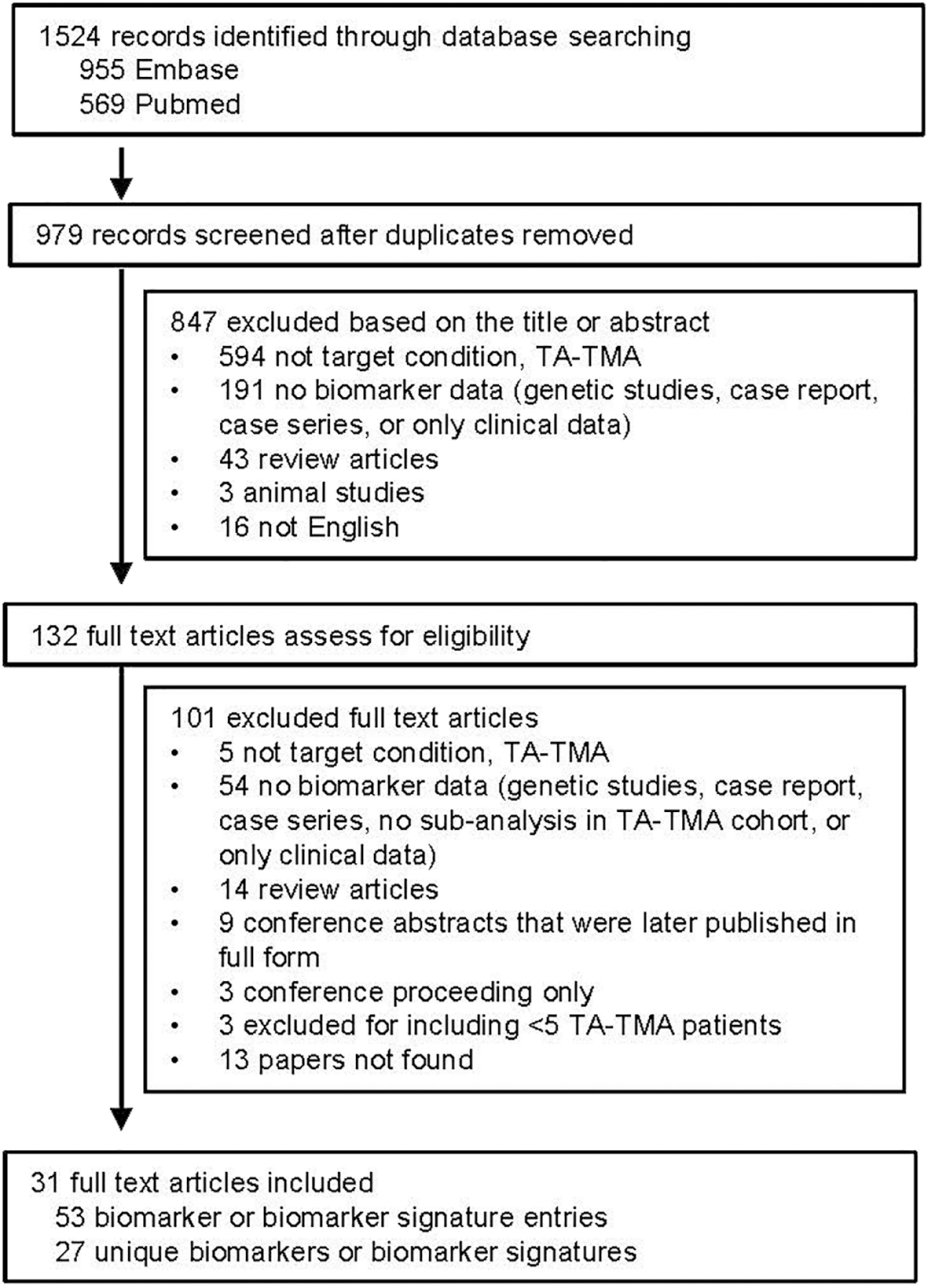
PRISMA flow chart of paper selection.

After removing duplicates, 979 publications were identified. After reviewing titles and abstracts, 132 were then manually reviewed. Most publications were excluded because they were not addressing TA-TMA or did not include biomarker data ( **Figure 1** ). A total of 53 biomarker entries were identified that included 27 unique biomarkers or biomarker signatures.

### Characteristics and quality of included studies

**Figure 2:**
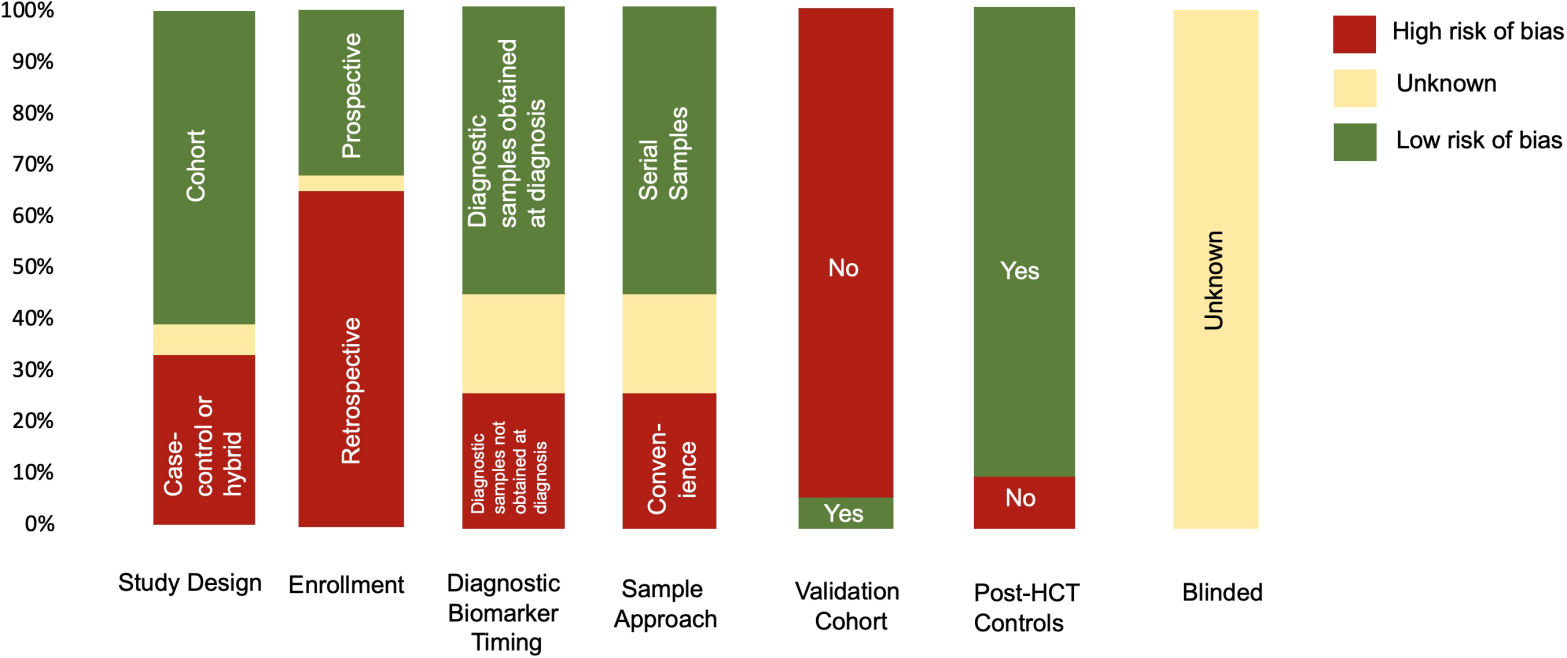
Summary of QUADAS-2 assessment for the risk of bias to assess study quality. Results of the QUADAS-2 questions for each study (n=31). If information was missing from the manuscript, it was indicated as unknown. In addition to typical QUADAS-2 questions, data for timing of diagnostic samples, validation cohort, and controls (whether the cohort was post HCT or healthy donors) are also indicated.

Summarized results for the quality and risk of bias assessments of the 31 studies based on QUADAS-2 are shown in **Figure 2**. The risk of bias was shown to be high in most studies given case–control study designs, convenience sample approaches, retrospective study design, and lack of validation in a separate cohort. No study commented on whether or not the samples were blinded to biomarker analysis.

### Entries per biomarker category and type

**Figure 3:**
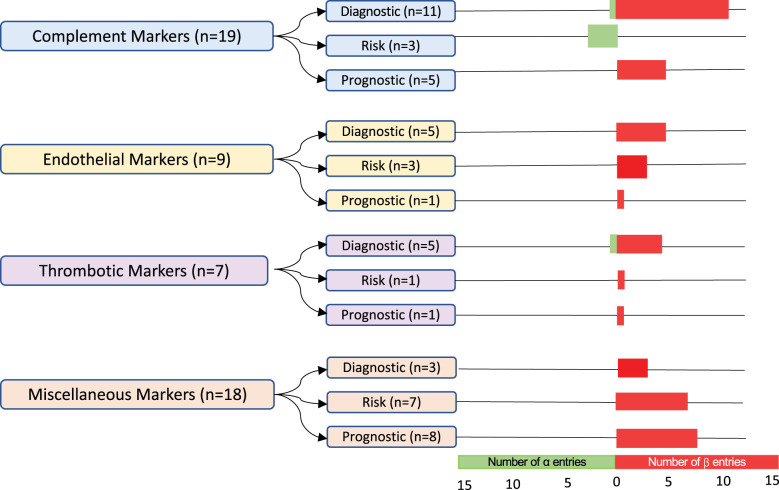
Classification and number of entries per biomarker category. In total, 53 biomarker entries from a set of 31 studies were included. The α-group entries included sensitivity and specificity values and post-HCT controls. The β-group entries lacked either or both of these features. Biomarkers were grouped into markers of complement activation, endothelial activation, thrombosis, and miscellaneous. They were further subdivided into diagnostic, risk, and prognostic biomarkers. The number of α-group entries is indicated in green, and the number of β-group entries is in red.

The 53 biomarker entries, from 31 studies, were classified into four predefined categories: markers of complement activation, endothelial activation, thrombosis, or other ( **Figure 3** ). Markers of complement activation comprised 36% of biomarker entries. Sample sizes of cohorts varied with a median TA-TMA sample size of 15 patients (range 7–508 patients) and a median control cohort size of 26 patients(range 3–1482 patients). Multiple different diagnostic criteria were used including Cho (n= 8), Jodele (n=5), the Bone Marrow Transplant Clinical Trials Network (BMT-CTN) (n=4), the International Working Group (IWG) (n=3), City of Hope (n=1), Uderzo (n=2), multiple criteria (n=3), and other/unknown (n=5). Fourteen (45%) of the studies were with adults, 12 (39%) were with children, three (10%) included both children and adults, and two (6%) did not report age.

### Biomarkers with reported diagnostic performance (α-group)

Of the five studies that reported a biomarker with sensitivity and specificity, two were diagnostic and three predicted later risk of TA-TMA. Levels of sC5b-9 ≥ 321.5 ng/ml in an adult cohort of 10 patients with TA-TMA and GVHD, compared with 10 patients with GVHD, only had a 90% sensitivity and a 70% specificity for TA-TMA diagnosis (28). However, this was a case–control study, limited by a small sample size, and it was not clear when samples were obtained. vWF% activity of ≥325% at the time of TA-TMA diagnosis had an 86.96% sensitivity [95% confidence interval (CI) 66.4% to 97.2%] and a specificity of 67.25% (95% CI 53.3% to 79.3%) (29). This prospective study had a larger sample size (TA-TMA, n=23; controls, n=53), but the findings were not validated in a separate cohort.

Two biomarkers were associated with the later development of TA-TMA. Levels of Ba ≥869.1 ng/ml on day 7 post HCT had a sensitivity of 80% (95% CI 52% to 96%) and a specificity of 100% (95% CI 70% to 100%) of predicting later development of TA-TMA (median day 25, range 22.5–44 days) (30). Although sensitivity and specificity were reported, this study was limited by a case–control study design and small sample size of 15 patients with TA-TMA and 15 post-HCT controls. The only biomarker that was validated in two separate cohorts was sC5b-9 level on day 28. In two pediatric cohorts, levels of sC5b-9 ≥252 ng/ml on day 28 had a sensitivity of 100% and a specificity of 61% in the first cohort, and in the validation group a sensitivity of 100% and a specificity of 53% for developing TA-TMA later in the HCT course (median day 63, range 42–85 days) (31, 32). Although validation was a strength of this study, the sample size was a limitation. Between both cohorts, only 20 patients had TA-TMA (10 in the first cohort and 10 in the second). The same group also reported that a change in 66 ng/ml from baseline to day 28 day had a sensitivity of 90% and a specificity of 84% of a later TA-TMA diagnosis (32) (**Table 2**).

**Table 2:** α-group biomarkers.

	Biomarker	Ref	Study (year)	TMA Dx criteria	Age	Control group	Study design	Enrollment	Sample timing	Blinding	Validation cohort	Size		Sensitivity (95% CI)	Specificity(95% CI)
TMA	Control		
Diagnostic	vWF ≥325%	(29)	Xu (2020)	Cho	A	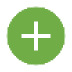	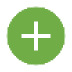	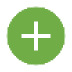	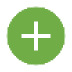	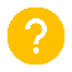	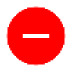	23	53	86.96% (66.4% to 97.2%)	67.25% (53.3% to 79.3%)
	sC5b-9 ≥ 321.5 ng/ml	(28)	Gavriilaki (2019)	IWG	A	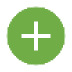	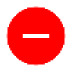	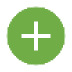	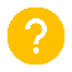	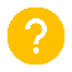	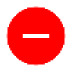	10	20 GVHD and Control	90%	70%
Predictive	Ba, ≥869.1 ng/ml	(30)	Okamura (2021)	Cho	A	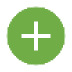	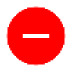	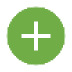	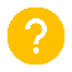 D 7	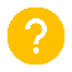	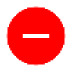	15	15	80% (52% to 96%)	100% (70% to 100%)
	sC5b-9, ≥252 ng/ml	(31) (32)	Horvath (2018) Mezo (2020)	IWG, Cho, Jodele, BMT-CTN, City of Hope	P P	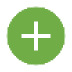	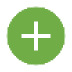	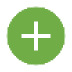	D28	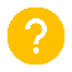	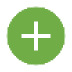	10 10	23 57	Training cohort, 100% Validation cohort, 100%	Training cohort, 61% Validation cohort, 53%
	sC5b-9, change from baseline to day 28 of 66 ng/ml	(32)	Mezo (2020)	IWG, Cho, Jodele, BMT-CTN, City of Hope	P	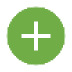	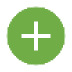	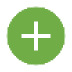	Day 28	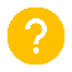	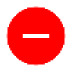	10	57	90%	84%

The α-group entries reported a sensitivity, specificity and included post-HCT controls. If the cohort was largely aged <18 years old, pediatrics (P) is indicated. If the cohort was adults (>18 years old), this i indicated with an A. All missing data are indicated with a 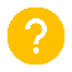, lowest biases with a 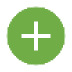, and higher bias risk with a 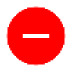. Low risk of bias by categories were defined as follows: any post-HCT controls, cohort study design, prospective study, serial enrollment of patients, samples obtained at the time of diagnosis for diagnostic criteria, and validation of biomarkers in a separate cohort. Otherwise, the study features were considered to have a high risk of bias.

diagnosis (dx).

#### β-group diagnostic biomarkers

Among β-group biomarker entries (n= 22), there were 13 unique biomarkers and one scoring system incorporating multiple biomarkers. Of the 14 diagnostic biomarkers, seven (50%) were obtained at the time of clinical diagnosis, five (32%) were obtained at a standard time point available, and the timing of the sample was unclear in two (14%). Markers of complement activation were significantly higher in patients with TA-TMA and included the alternative pathway, Ba (n=1) (33) and Bb (n=1) (34), the classical pathway, CH50 (n=1) (35), terminal complement, sC5b-9 (n= 4) (2, 34–36), and anaphaylatoxin C3b (n=1). Markers of endothelial activation, including ST2 (n=2) (9, 37), sVCAM-1 (n=2) (28, 38), and ANG-2 (n=1) (36), markers of thrombosis, including thrombomodulin (n=3) (28, 37, 39), vWF : Ag (39), neutrophilic extracellular traps measured by dsDNA, and day 100 endothelial activation, and stress index (EASIX) scores, were calculated (lactate dehydrogenase (LDH) x serum creatinine)/platelets. These markers were also significantly higher in TA-TMA patients (40). Levels of heme oxygenase-1, a stress-inducible protein, which protects endothelial cells from apoptosis, were lower in patients with TA-TMA than in post-HCT controls (41) ( **Table 3** ).

**Table 3:** β-group diagnostic biomarkers.

Category	Biomarker	Ref	Study (year)	TMA Dx criteria	Age range	Control group	Study design	Enrollment	Serial samples	Sample timing	Validation cohort	Blinding	Size	
TMA	Controls
Complement	sC5b-9	(15)	Jodele (2014)	Jodele	P	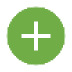	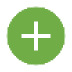	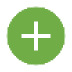	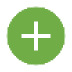	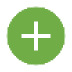	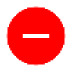	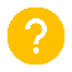	39	20
	sC5b-9	(35)	Qi (2017)	Cho	A	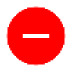	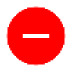	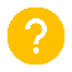	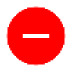	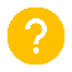	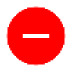	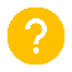	20	74 self-BL
	sC5b-9	(34)	Wall (2018)	Wall	A	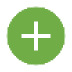	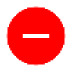	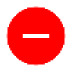	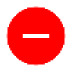	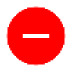 **	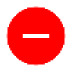	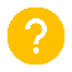	29 TMA and GVHD	12 GVHD
	sC5b-9	(36)	Li (2020)	Li	A	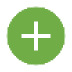	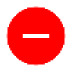	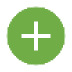	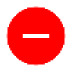	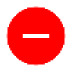	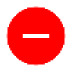	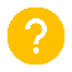	12 TMA and GVHD	24 GVHD
	sC5b-9	(30)	Gavriilaki (2019)	IWG	A	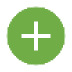	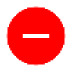	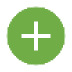	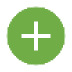	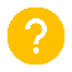	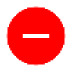	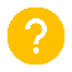	10	10
	C3b	(35)	Qi (2017)	Cho	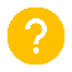	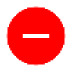	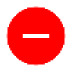	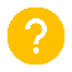	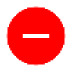	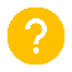	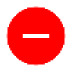	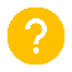	20	74 self-BL
	mHam	(30)	Gavriilaki (2019)	IWG	A	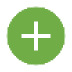	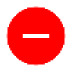	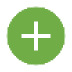	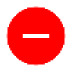	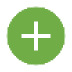	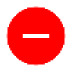	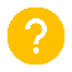	10	20 GVHD and control
	CH50	(35)	Qi (2017)	Cho	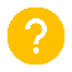	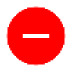	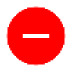	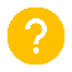	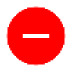	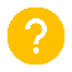	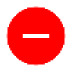	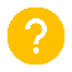	20	74 self-BL
	Ba	(33)	Sartain (2019)	Baylor	P	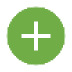	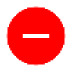	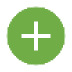	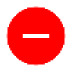	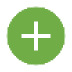	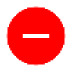	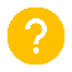	7	7
	Bb	(34)	Wall (2018)	Wall	A	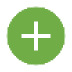	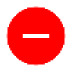	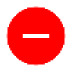	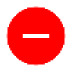	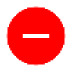 **	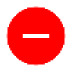	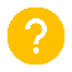	29 TMA and GVHD	12 GVHD
Endothelial	ST2	(9)	Vasu (2022)	Cho	A	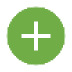	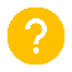	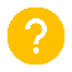	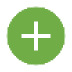	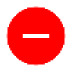 **	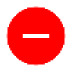	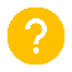	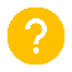	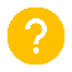
	ST2	(37)	Zeisbrich (2017)	IWG	A	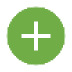	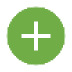	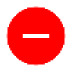	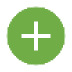	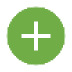	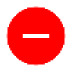	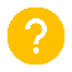	12	18 CKD
	sVCAM-1	(30)	Gavriilaki (2019)	IWG	A	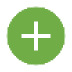	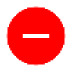	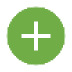	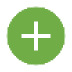	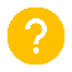	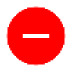	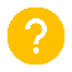	10	10
	sVCAM-1	(38)	Matsuda (2001)	BMT-CTN	P	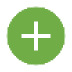	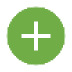	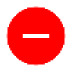	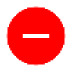	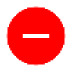 D30	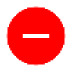	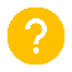	7	11
	ANG2	(36)	Li (2020)	Li	A	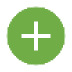	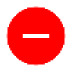	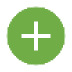	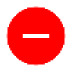	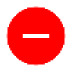 **	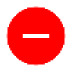	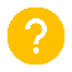	12 TMA and GVHD	24 GVHD
Thrombotic	vWF : Ag	(42)	Zeigler (1996)	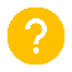	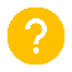	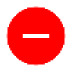	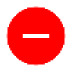	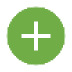	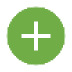	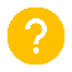	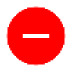	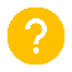	39	20 healthy controls
	TM	(42)	Zeigler (1996)	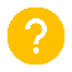	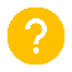	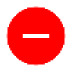	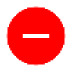	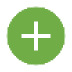	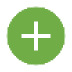	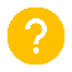	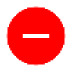	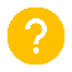	39	27 TTP
	TM	(37)	Zeisbrich (2017)	IWG	A	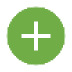	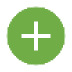	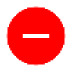	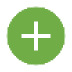	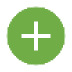	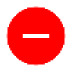	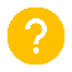	12	18
	TM	(30)	Gavriilaki (2019)	IWG	A	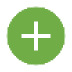	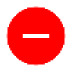	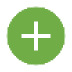	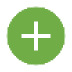	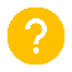	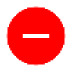	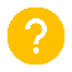	10	10
Miscellaneous	Heme oxygenase-1	(41)	Pan (2019)	Cho	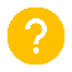	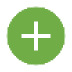	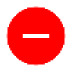	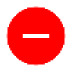	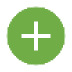	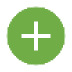	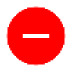	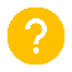	15	45
	Day 100 EASIX score	(43)	Gavriilaki (2021)	IWG	A	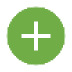	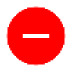	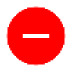	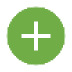	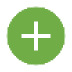	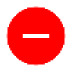	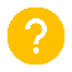	20 TMA and GVHD	20 post-HCT controls
	dsDNA (NETs)	(40)	Arai (2013)	BMT-CTN	A	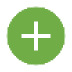	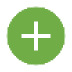	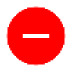	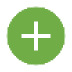	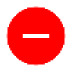 D30	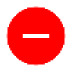	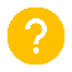	11	79

The β-group entries lacked sensitivity or specificity. Th age range of patients in the study is indicated if included, otherwise based on median and interquartile ranges provided; if the cohort was largely aged <18 years, pediatrics (P) is indicated. All missing data are indicated with a 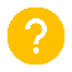, lowest biases with a 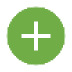, and higher bias risk with a 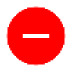. Low risk of bias by categories were defined as follows: any post-HCT controls, cohort study design, prospective study, serial enrollment of patients, samples obtained at the time of diagnosis for diagnostic criteria, and validation of biomarkers in a separate cohort. Otherwise, the study features were considered to have a high risk of bias. **Indicates the samples were obtained at the time of GVHD diagnosis in patients with concurrent TA-TMA and GVHD.

modified HAM (mHAM), thrombomodulin (TM), endothelial activation and stress index (EASIX), double stranded DNA (dsDNA), thrombocytopenic thrombotic purpura (TTP), baseline (BL).

#### β-group risk biomarkers

Eleven biomarker entries from 10 studies resulted in eight unique biomarkers, which were significantly different before clinical manifestations of TA-TMA and may predict the development of the disease. Timing of samples acquisition varied significantly: six studies (55%) measured samples at baseline (BL) preconditioning, three (27%) on day 14 post HCT, one (9%) 2 weeks before clinical TA-TMA diagnosis, and one (9%) a range of days before TA-TMA diagnosis (median 37 days). There were no β-group risk markers of complement activation. ST2 was the most promising risk biomarker. It was significantly elevated in patients with TA-TMA, compared with controls, before clinical manifestations of the disease in three separate studies (9, 37, 44). Early elevation of dsDNA, thought to be from neutrophilic extracellular traps, was significantly elevated in TA-TMA patients in two studies (40, 45) (**Table 4** ).

**Table 4:** β-group risk biomarkers.

Category	Biomarker	Ref	Study (year)	TMA Dx criteria	Age	Control group	Study design	Enrollment	Serial samples	Sample timing	Validation cohort	Blinding	Size	
TMA	Controls
Endothelial	ST2	(9)	Vasu (2022)	Cho	A	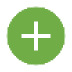	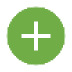	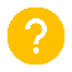	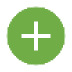	BL	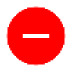	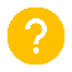	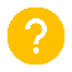	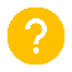
	ST2 >1180 ng/dl	(37)	Zeisbrich (2017)	IWG	A	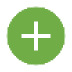	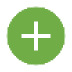	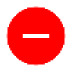	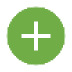	BL	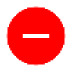	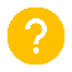	12	18 CKD
	ST2	(44)	Rotz (2017)	Jodele	P	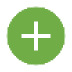	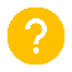	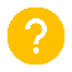	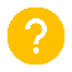	D14	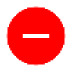	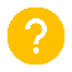	115	194
Thrombotic	vWF %	(29)	Xu (2019)	Cho	A	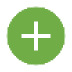	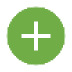	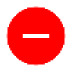	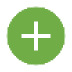	37 days before TMA dx	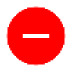	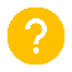	23	56
Miscellaneous	dsDNA (NETS)	(45)	Gloude (2017)	Jodele	P	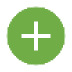	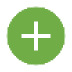	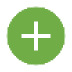	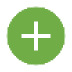	D14	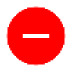	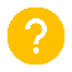	37	66
	dsDNA (NETS)	(40)	Arai (2013)	BMT-CTN	A	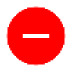	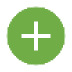	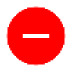	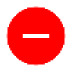	BL	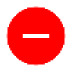	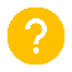	11	10 healthy controls
	Insulin-like growth factor-1	(46)	Betzmann (2022)	BMT-CTN	P	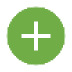	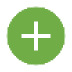	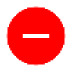	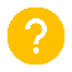	BL	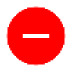	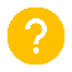	8	490
	F-actin	(47)	Luebbering (2021)	Jodele	P	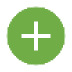	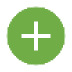	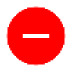	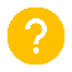	D14	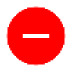	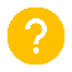	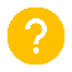	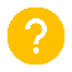
	Baseline LDH	(48)	Postalcioglu (2018)	City of Hope	A	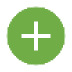	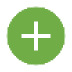	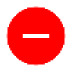	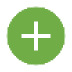	BL	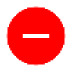	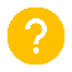	508	1482
	Haptoglobin degradation product	(49)	Schuh (2019)	Cho	P	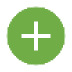	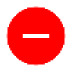	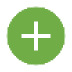	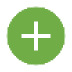	2 wks before TMA dx	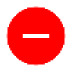	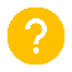	13	3
	Nitrates	(37)	Zeisbrich (2017)	IWG	A	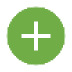	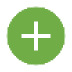	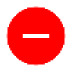	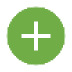	BL	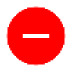	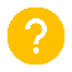	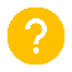	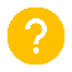

The β-group entries lacked sensitivity or specificity. The age range of patients in the study is indicated if included, otherwise based on median and interquartile ranges provided; if the cohort is largely aged <18 years, P (pediatrics) is indicated. All missing data are indicated with a 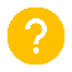, lowest biases with a 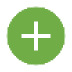, and higher bias risk with a 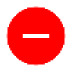. Low risk of bias by categories were defined as follows: any post-HCT controls, cohort study design, prospective study, serial enrollment of patients, samples obtained at the time of diagnosis for diagnostic criteria, and validation of biomarkers in a separate cohort. Otherwise, the study features were considered to have a high risk of bias.

thrombomodulin (TM), endothelial activation and stress index (EASIX), double stranded DNA (dsDNA), thrombocytopenic thrombotic purpura (TTP), baseline (BL), chronic kidney disease (CKD).

#### β-group prognostic biomarkers

Eleven biomarkers associated with poor prognosis were reported. Outcomes included death, non-relapse mortality, and neurological dysfunction (50). Four studies reported prognostic implications of sC5b-9 including (1) values ≥ 244 ng/dl at the time of diagnosis (2); (2) a change in level of 1.8-fold from baseline to diagnosis (51); (3) an increase of a log at the time of TA-TMA (51); and (4) levels of sC5b-9 ≥300 ng/l and ANG-2 > 3000 pg/ml in patients with concurrent TA-TMA and GVHD (36). Markers of microangiopathy including decreased levels of haptoglobin (52), elevated levels of LDH (15, 53), and scores that use laboratory values of microangiopathy, including BATAP (bilirubin, age, thrombocytopenia, anemia, proteinuria) (54) TMA index (LDH/platelets), and an EASIX score (55), all portended a poor prognosis. also portended a poor prognosis (14). BATAP scores and their association with mortality were studied in two independent cohorts and validated at the time of TA-TMA diagnosis in a large cohort. Of note is that the only identified positive prognostic biomarker was red cell fragments, which when present, compared with schistocytes without red cell fragments, was associated with decreased mortality (56) ( **Table 5** ).

**Table 5:** β-group prognostic biomarkers.

Category	Biomarker	Ref	Study (year)	TMA Dx criteria	Age	Control group	Study design	Enrollment	Serial samples	Sample timing	Validation	Blinding	Size	
Cases	Controls
Complement	sC5b-9 ≥ 244 ng/dl	(2)	Jodele (2014)	Jodele	P	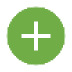	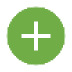	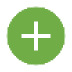	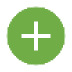	TMA dx	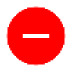	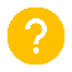	18 TA-TMA dead	18 TA-TMA alive
	sC5b-9	(30)	Gavriilaki (2019)	IWG	A	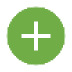	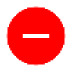	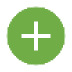	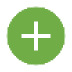	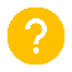	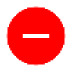	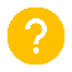	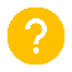 Dead	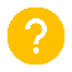 Alive
	sC5b-9 Δ from BL to diagnosis 1.8-fold	(51)	Jodele (2022)	Jodele	P	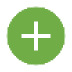	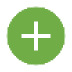	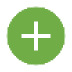	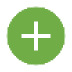	TMA dx	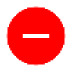	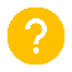	18 MR TA-TMA dead	30 MR TA-TMA alive
	Log-sC5b-9	(51)	Jodele (2022)	Jodele	P	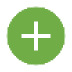	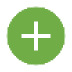	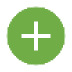	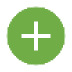	TMA dx	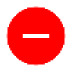	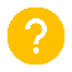	18 MR TA-TMA dead	30 MR TA-TMA alive
	Ba ≥ 869.1 ng/ml	(32)	Okamura (2021)	Cho	A	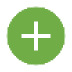	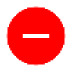	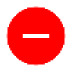	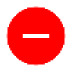	D 7	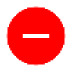	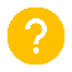	12 High Ba	18 Low Ba
Endothelial	sC5b-9 ≥300 ng/dl and ANG-2 >3000 pg/ml	(36)	Li (2020)	Li	A	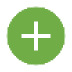	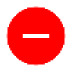	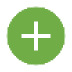	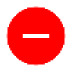	**	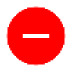	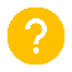	12 TA-TMA and GVHD	24 acute GVHD
Thrombotic	vWF % ≥ 325%	(31)	Xu (2019)	Cho	A	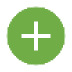	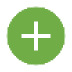	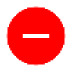	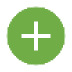	TMA dx	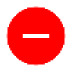	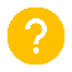	38 ≥ 325% vWF%	40 <325% vWF%
Miscellaneous	Calpain Activity	(50)	Zeigler (1999)	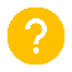	A	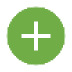	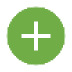	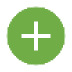	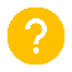	TMA dx	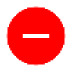	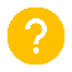	19 TA-TMA, no neurological change	3 TA-TMA neurological changes
	Proteinuria ≥ 30 mg/dl	(2)	Jodele (2014)	Jodele	P	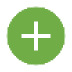	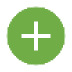	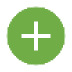	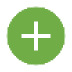	TMA dx	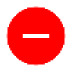	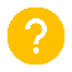	18 TA-TMA dead	18 TA-TMA Alive
	Haptoglobin below normal limits	(52)	Zhang (2018)	Cho	P and A	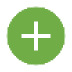	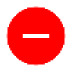	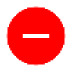	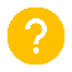	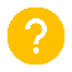	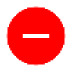	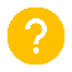	24 TA-TMA dead	26 TA-TMA alive
	Peak LDH	(47)	Uderzo (2000)	Other	P	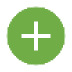	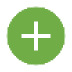	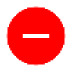	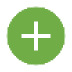	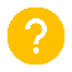 Max LDH	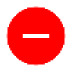	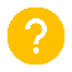	9 severe TA-TMA	19 non-severe TA-TMA
	LDH at TA-TMA diagnosis	(15)	Schoettler (2020)	Jodele	P	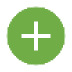	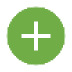	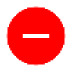	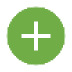	TMA dx	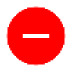	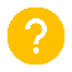	50 TA-TMA dead	50 TA-TMA alive
	Red cell fragments*	(49)	Jekarl (2015)	BMT-CTN	A	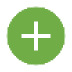	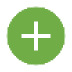	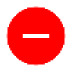	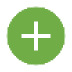	TMA dx	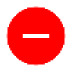	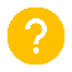	15 TA-TMA	74 no TA-TMA
	BATAP score	(54)	Zhao (2021)	Cho	P and A	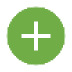	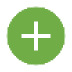	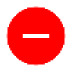	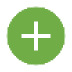	TMA dx	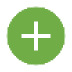	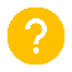	223 TA-TMA dead	285 TA-TMA, alive
	TMA index (LDH/platelets)	(14)	Uderzo (2006)	Other	P and A	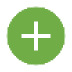	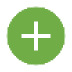	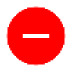	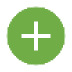	TMA dx	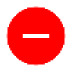	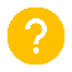	32 TA-TMA dead	32 TA-TMA alive

The β-group entries lacked sensitivity or specificity. The age range of patients in the study was indicated if included, otherwise based on median and interquartile ranges provided; if the cohort was largely aged <18 years, P (pediatrics) is indicated. All missing data are indicated with a 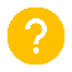, lowest biases with a 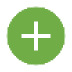, and higher bias risk with a 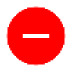. Low risk of bias by categories were defined as follows: any post-HCT controls, cohort study design, prospective study, serial enrollment of patients, samples obtained at the time of diagnosis for diagnostic criteria, and validation of biomarkers in a separate cohort. Otherwise, the study features were considered to have a high risk of bias. The BATAP score was comprised of 1 point each for bilirubinemia, age, thrombocytopenia, anemia, and proteinuria. Moderate risk TA-TMA (MR) was defined as elevated sC5b-9 or random urine protein-to-creatinine ratio without evidence of multi-organ dysfunction. All markers are associated with a poor prognosis, either non-relapsed mortality or death except red cell fragments (*). The presence of red cell fragments in this study was a positive prognostic factor associated with improved survival. ** sample obtained at GVHD diagnosis

thrombomodulin (TM), double stranded DNA (dsDNA), thrombocytopenic thrombotic purpura (TTP), baseline (BL), moderate risk TA-TMA (MR TA-TMA).

## Discussion

This systematic review assessed the diagnostic, predictive, and prognostic performance of biomarkers of TA-TMA at multiple time points. A wide variety of biomarkers were tested, but only three biomarkers had specificity and sensitivity data and only two were validated in separate cohorts. These studies had the strongest level of evidence in this systematic review, but there were still limitations due to the small sample size and study design. The single biomarker with the most robust data is sC5b-9 levels, which has reported diagnostic, risk, and prognostic associations. Furthermore, the sC5b-9 level was one of only two biomarkers that was confirmed in a separate validation cohort. However, there is insufficient evidence that elevated levels of sC5b-9 alone are specific for TA-TMA. Furthermore, the appropriate cut-off value for the sC5b-9 level is not clear, with contradictory data in children and adults. Lastly, the sC5b-9 level is not readily available, limiting its incorporation into clinical practice. Outside of CH50, LDH, blood counts, and proteinuria, most tests are only obtained in specialty laboratories and, hence, not readily available. Furthermore, although some studies reported significant differences in sC5b-9 kinetics these have limited clinical utility at this time, as these biomarkers are not routinely assessed before the transplant or serially post transplant.

### Gaps identified from review in current literature and future directions

This review summarizes the biomarker landscape of TA-TMA and highlights the many unmet needs in the field. (1) There are very low-bias risk studies, and many are limited by small sample size, with a median of 15 patients with TA-TMA. (2) Many biomarkers do not have a normal range of concentration or level established. Even if a normal range is established, in the post-HCT setting the normal range can likely not be applied. Thus, a cut-off value of biomarkers is necessary for clinical utility. Only five studies reported a biomarker with a cut-off value and associated sensitivity and specificity. (3) There was significant heterogeneity in TMA diagnostic criteria used among studies. As some criteria incorporate organ dysfunction, this may bias results. (4) Among diagnostic biomarkers, only 50% were obtained at the time of clinical diagnosis. Utilizing institutional biorepositories with samples available at standard time points is often more feasible than collecting samples at the time of the event, but when investigating diagnostic biomarkers, obtaining samples at the time of the event is critical and the most rigorous approach. (5) A lack of validation in separate cohorts and demonstration of reproducibility of results. Most markers are not clinically available and, in most studies, enzyme-linked immunosorbent assay (ELISA) kits were used from multiple different companies (**Supplement Table 3**). We have made suggestions to address these gaps for future research (**Table 6**).

**Table 6:** Key findings and suggestions for future studies.

Topic area	Key findings	Suggestions for future work
TA-TMA Diagnostic Criteria	- Multiple different criteria continue to be used to diagnose TA-TMA. This may bias results, with some cohorts being more ill at the time of diagnosis and makes applicability of study results in different populations challenging.	- As per consensus recommendation, Jodele et al criteria should be used for diagnosis of TA-TMA.
Sample size	- Sample sizes, particularly of TA-TMA cases were small limiting power.	- Development of large multi-institutional bio-banking efforts in patients with diversity in age, disease indication, and transplant characteristics who are universally screened for TA-TMA with rich linked clinical data.
High prevalence of concurrent complications/ Selection of controls.	- TA-TMA often occurs with GVHD, viral infection, and VOD as shown in **Figure 4**. - These complications have overlapping biomarkers.	Large studies with approaches to adjust for concurrent comorbidity including perhaps 1) matching controls with same complications or 2) novel groups including TA-TMA, GVHD, TA-TMA & GVHD, and neither. “sub in whatever complication” time of TA-TMA event
Age	- Paucity of data specifically within extreme ages ranges; the very young <12 months and elderly >70 years of age.	- TA-TMA biomarker studies including a wide range of ages.
Selection of endpoints for prognosis	- End points for prognostic markers varied included severe disease (i.e. neurologic manifestations), non-relapse mortality, and death.	- Given the complexity of these patients, organ failure or non-relapse related mortality (NRM) are preferred over all-cause mortality.
Timing of sample collection	- For diagnostic biomarkers, samples were often not collected at the time of event, and instead investigators relied on standard time points with available samples. - Predictive and prognostic biomarkers were reported in early time points (like pre-HCT and day 7) regardless of time of TA-TMA diagnosis.	- Diagnostic biomarkers should be tested at the time of event when possible. - Predictive and prognostic biomarkers at multiple standard time points like day 30, 60, 100, and 180 may allow investigation of both early and late onset TA-TMA.
Study Design	- Many studies were case control, retrospective, and/or included convenience sampling—all study design features with higher risks of bias.	- Prospective studies with serial sample collection are needed to minimize bias.
Sources of biomarkers	- Most data reviewed were blood biomarkers. While some immunohistochemistry studies of tissue staining were excluded, no other sources of biomarkers were identified.	- Collecting other fluid sources including urine, bronchoscopy fluid, etc may yield additional data, particularly regarding the tropism of organ dysfunction seen in TA-TMA.
Biomarker panels versus single markers	- There were some scoring systems used incorporating multiple markers including BATAP, EASIX and the TMA-index, but most biomarker studies looked at single markers.	- Panels of biomarkers are likely to be most sensitive and specific for TA-TMA both when it occurs alone and with concurrent comorbidities. - Collaborative efforts to allow multiple groups to work on biomarker discovery and combining biomarkers are needed.
Reporting	- In many studies, it is difficult to find key information including study design, timing of samples, patient recruitment, and the number of samples used, which often differed from the number of patients and characteristics described in Table 1. - Blinding of sample analysis was not reported in any study.	- Following STROBE, STARD, or CONSORT checklists as relevant will ensure information is included in publications.
Discovery studies versus validation and confirmatory studies	- The performance of many biomarkers is measured only with a p-value, and studies are often underpowered. - There were only 2 studies in all of the literature reviewed that included a validation cohort. - AUC curves are reported, but no cut-offs, limiting the clinical utility of biomarkers tested.	- Biomarker studies should have separate discovery and validation cohorts. - Cut off values should be determined in a discovery cohort and then biomarkers tested in the validation cohort. AUCS should be reported in both cohorts, as well as the sensitivity and specificity of the cut point determined in the discovery cohort.
Clinical Utility	- Most biomarkers tested are not readily available clinically. - If tests are available, the appropriate cut off in the post-HCT setting is not known.	- Increase the availability of complement and endothelial activation testing.

**Figure 4:**
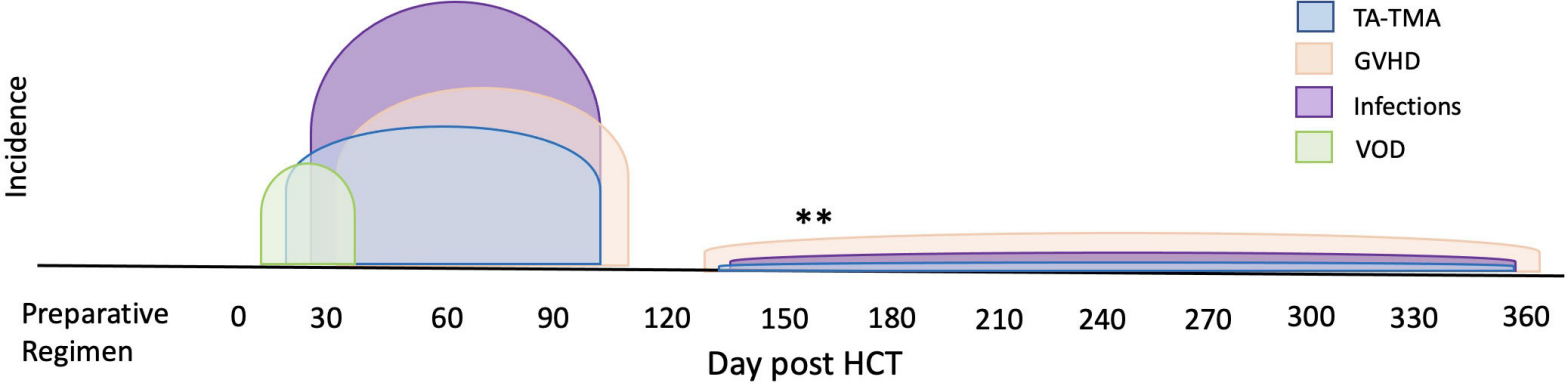
Overlap of TA-TMA and common post-BMT complications in time. The typical high-risk period for complications post-HCT are indicated in different colors. The high-risk period for TA-TMA overlaps with the risk period for acute GVHD, VOD, infections, and organ toxicities such as pulmonary failure. **Often in the setting of overlapping or chronic GVHD, increased risks for infections and TA-TMA can occur later in the transplant course.

**Figure 5:**
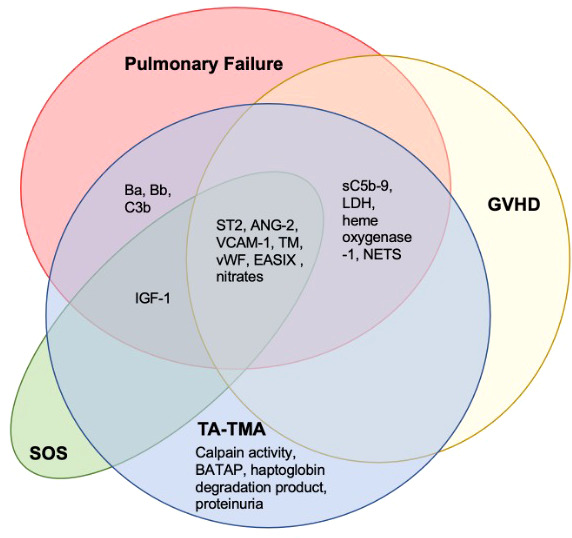
Overlapping biomarkers of common post-BMT complications and TA-TMA. A literature search of the TA-TMA biomarkers identified in this review and any diagnostic, prognostic, or risk implications in GVHD, SOS, and pulmonary failure (infectious and non-infectious) was completed. Most biomarkers identified in TA-TMA overlapped with other post-HCT complications. Citations provided in **Supplementary Table 2**. Abbreviations: lactate dehydrogenase (LDH), neutrophil extracellular traps (NETS), thrombomodulin (TM).

Another pervasive challenge of biomarker discovery in TA-TMA is concurrent comorbidities—TA-TMA often occurs with acute GVHD, hepatic sinusoidal obstructive syndrome, and/or infections ( **Figure 4** ). Each of these complications has reported biomarkers, many of which overlap with the biomarkers reported in TA-TMA ( **Figure 5** ). This challenge is not unique to TA-TMA and impacts most biomarker research in HCT. Because GVHD and infections, well-known risk factors for TA-TMA can directly cause endothelial damage (42, 43, 57, 58), they may play a key role in the pathophysiology of TA-TMA. Thus, adjusting for these complications in a typical multivariable biomarker analysis may not be the best approach (59). Instead, to overcome this limitation, biomarkers of TA-TMA could have three separate comparison groups: (1) post HCT without any complications, (2) TA-TMA with a concomitant complication of interest (i.e., GVHD, steroid-refractory GVHD, SOS, infections, etc.), and (3) only complication of interest without TA-TMA. This approach could help overcome this limitation and gain insight into the shared and disparate biomarkers of two diseases. However, this would require large sample sizes and multi-institutional collaboration. Furthermore, because multiple complications are often present this cannot resolve all limitations. Very large cohorts, with controls enriched for concurrent morbidities using other approaches like matching or propensity score matching, is another approach that could be taken.

Based on our systemic review, while many candidate biomarkers were identified, there is insufficient sensitivity and specificity data for a single biomarker or biomarker panel to be used for the diagnosis of TA-TMA. Continued efforts to identify diagnostic, prognostic, and risk biomarkers in TA-TMA and validate them in separate cohorts is a priority for this disease. Biomarker discovery is critical for clinical care and may also provide additional insight into the pathophysiology of the disease and support novel therapeutic approaches. There are currently inhibitors that target multiple components of the complement cascade, including the lectin pathway, the alternative pathway, the classical pathway, C3, and C5 (7, 60, 61). Evidence of activation in any of these pathways could support investigating the safety and efficacy of these novel therapeutic approaches. We propose a continued investigation of biomarkers, with the most robust data to date including markers of complement activation, ST2, and vWF % and markers that could have therapeutic implications, or which are readily accessible.

### Strengths and limitations of this study

This systematic review outlined how the results of 31 papers and 27 unique biomarkers have several strengths. Inclusion and exclusion criteria were defined and agreed on by authors a priori. The search strategy was validated and PRISMA guidelines were followed. Quality and bias assessment of publications were assessed using QUADAS-2, a recognized and validated tool. There were no limitations on patient populations: children and adults were included using any TA-TMA diagnostic criteria. However, the review was limited by a lack of formal assessment of publication bias. We could not perform a meta-analysis given the heterogeneity of the studies. We included only studies written in English, and may have missed studies written in other languages, though we are not aware of any such misses. Furthermore, we could have missed other biases that were not covered by the QUADAS-2 questions. Lastly, we reported only “positive” results and did not summarize contradictory studies in which there were no significant differences in biomarkers.

## Conclusions

The increased interest and recognition of TA-TMA is exciting, but for the field to move forward and make significant, clinically applicable progress, multi-institutional, collaborative efforts with samples obtained at appropriate time points with well-matched controls are needed. We conclude that currently, while there is promising early data, more robust evidence with large validation cohorts are needed for diagnostic, prognostic, and predictive biomarkers in TA-TMA.

## Author contributions

MS and SV designed and wrote and manuscript, MS and HB reviewed articles with the oversight of MS and SV. All authors edited the manuscript and approved the final version.

## References

[1] Gavriilaki E Sakellari I Batsis I Mallouri D Bousiou Z Vardi A. Transplant-associated thrombotic microangiopathy: Incidence, prognostic factors, morbidity, and mortality in allogeneic hematopoietic cell transplantation. Clin Transplant. (2018) 32(9):e13371. doi: 10.1111/ctr.13371 30080283

[2] Jodele S Davies SM Lane A Khoury J Dandoy C Goebel J. Diagnostic and risk criteria for HSCT-associated thrombotic microangiopathy: a study in children and young adults. Blood. (2014) 124(4):645 LP – 653. 10.1182/blood-2014-03-564997 PMC4110664 24876561

[3] Jodele S Dandoy CE Myers K Wallace G Lane A Teusink-Cross A. High-dose Carboplatin/Etoposide/Melphalan increases risk of thrombotic microangiopathy and organ injury after autologous stem cell transplantation in patients with neuroblastoma. Bone Marrow Transplant. (2018). doi: 10.1038/s41409-018-0159-8 29674658

[4] Young JA Pallas CR Knovich MA. Transplant-associated thrombotic microangiopathy: theoretical considerations and a practical approach to an unrefined diagnosis. Bone Marrow Transplant. (2021) 56(8):1805–17. doi: 10.1038/s41409-021-01283-0 PMC8338557 33875812

[5] Jodele S Sabulski A. Transplant-associated thrombotic microangiopathy: elucidating prevention strategies and identifying high-risk patients. Expert Rev Hematol (2021) 14(8):751–63. doi: 10.1080/17474086.2021.1960816 34301169

[6] Dvorak CC Higham C Shimano KA. Transplant-associated thrombotic microangiopathy in pediatric hematopoietic cell transplant recipients: A practical approach to diagnosis and management. Front Pediatr . (2019) 7:133. 31024873 10.3389/fped.2019.00133 PMC6465621

[7] Schoettler M Chonat S Williams K Lehmann L. Emerging therapeutic and preventive approaches to transplant-associated thrombotic microangiopathy. Curr Opin Hematol (2021) 28(6). 10.1097/MOH.0000000000000687 PMC9908033 34534983

[8] Elfeky R Lucchini G Lum S-H Ottaviano G Builes N Nademi Z. New insights into risk factors for transplant-associated thrombotic microangiopathy in pediatric HSCT. Blood Adv (2020) 4(11):2418–29. doi: 10.1182/bloodadvances.2019001315 PMC7284098 32492158

[9] xVasu S Bostic M Zhao Q Sharma N Puto M Knight S. Acute GVHD BK virus hemorrhagic cystitis and age are risk factors for transplant-associated thrombotic microangiopathy in adults. Blood Adv (2022) 6(4):1342–9. doi: 10.1182/bloodadvances.2021004933 PMC8864665 34932790

[10] Daly AS Hasegawa WS Lipton JH Messner HA Kiss TL. Transplantation-associated thrombotic microangiopathy is associated with transplantation from unrelated donors, acute graft-versus-host disease and venoocclusive disease of the liver. Transfus Apher Sci (2002) 27(1):3–12. doi: 10.1016/S1473-0502(02)00020-4 12201469

[11] Schoettler M Stenger EO Spencer K Lutterman D Rumbika S Jones JA. Sickle cell disease is a risk factor for transplant-associated thrombotic microangiopathy in children. Blood Adv (2022). doi: 10.1182/bloodadvances.2022008058 PMC10164784 36075028

[12] Jodele S Zhang K Zou F Laskin B Dandoy CE Myers KC. The genetic fingerprint of susceptibility for transplant associated thrombotic microangiopathy. Blood. (2015) 127(8):989–97. doi: 10.1182/blood-2015-08-663435 PMC4828073 26603840

[13] Millicent E Gianluigi R Giulia A Valentina P Mariotti J Verna M. Gene abnormalities in transplant associated- thrombotic Microangiopathy: Comparison between recipient and donor ‘ s DNA. Thromb Haemost. (2021) 122(07):1247–50. 10.1055/s-0041-1740498 34965590

[14] Uderzo C Bonanomi S Busca A Renoldi M Ferrari P Iacobelli M. Risk factors and severe outcome in thrombotic microangiopathy after allogeneic hematopoietic stem cell transplantation. Transplantation. (2006) 82(5):638–44. doi: 10.1097/01.tp.0000230373.82376.46 16969286

[15] Schoettler M Lehmann LE Margossian S Lee M Kean LS Kao P-C. Risk factors for transplant-associated thrombotic microangiopathy and mortality in a pediatric cohort. Blood Adv (2020) 4(11):2536–47. doi: 10.1182/bloodadvances.2019001242 PMC7284101 32516415

[16] Li A Wu Q Davis C Kirtane KS Pham PD Sorror ML. Transplant-associated thrombotic microangiopathy is a multifactorial disease unresponsive to immunosuppressant withdrawal. Biol Blood Marrow Transplant. (2019) 25(3):570–6. doi: 10.1016/j.bbmt.2018.10.015 PMC6450411 30940363

[17] Jodele S Fukuda T Mizuno K Vinks AA Laskin BL Goebel J. Variable eculizumab clearance requires pharmacodynamic monitoring to optimize therapy for thrombotic microangiopathy after hematopoietic stem cell transplantation. Biol Blood Marrow Transplant. (2016) 22(2):307–15. doi: 10.1016/j.bbmt.2015.10.002 PMC4716886 26456258

[18] Jodele S Dandoy CE Lane A Laskin BL Teusink-Cross A Myers KC. Complement blockade for TA-TMA: lessons learned from a large pediatric cohort treated with eculizumab. Blood. (2020) 135(13):1049–57. doi: 10.1182/blood.2019004218 PMC7099329 31932840

[19] Yeates L Slatter MA Bonanomi S Lim FLWI Ong SY Dalissier A. Use of defibrotide to treat transplant-associated thrombotic microangiopathy: A retrospective study of the paediatric diseases and inborn errors working parties of the European society of blood and marrow transplantation. Bone Marrow Transplant. (2017) 52(5):762–4. doi: 10.1038/bmt.2016.351 28092354

[20] Khaled SK Claes K Goh YT Kwong YL Leung N Mendrek W. Narsoplimab, a mannan-binding lectin-associated serine protease-2 inhibitor, for the treatment of adult hematopoietic stem-cell transplantation–associated thrombotic microangiopathy. J Clin Oncol (2022) 40(22):2447–57. doi: 10.1200/JCO.21.02389 PMC9467678 35439028

[21] Ruutu T Barosi G Benjamin RJ Clark RE George JN Gratwohl A. Diagnostic criteria for hematopoietic stem cell transplant-associated microangiopathy: results of a consensus process by an international working group. Haematologica. (2007) 92(1 SE-Decision Making and Problem Solving):95–100. doi: 10.3324/haematol.10699 17229640

[22] Shayani S Palmer J Stiller T Liu X Thomas SH Khuu T. Thrombotic microangiopathy associated with sirolimus level after allogeneic hematopoietic cell transplantation with Tacrolimus/Sirolimus-based graft-versus-Host disease prophylaxis. Biol Blood Marrow Transplant. (2013) 19(2):298–304. doi: 10.1016/j.bbmt.2012.10.006 23078784 PMC3589900

[23] Uderzo C Jodele S El Missiry M Cicerci F Busca A Bacigalupa A CS. Transplant-associated thrombotic microangiopathy (TA-TMA) and consensus based diagnostic and therapeutic recommendations: Which TA-TMA patients to treat and when? J Bone Marrow Res (2014) 02(03). doi: 10.4172/2329-8820.1000152

[24] Levine JE Harris AC Taylor A Braun TM Magenau J Ferrara JLM. A biomarker-based grading system At onset of GvHD predicts NRM better than the modified glucksberg grading system. Blood. (2013) 122(21):145. doi: 10.1182/blood.V122.21.145.145

[25] Akil A Zhang Q Mumaw CL Raiker N Yu J Velez de Mendizabal N. Biomarkers for diagnosis and prognosis of sinusoidal obstruction syndrome after hematopoietic cell transplantation. Biol Blood Marrow Transplant. (2015) 21(10):1739–45. doi: 10.1016/j.bbmt.2015.07.004 PMC4568166 26172478

[26] Whiting PF Rutjes AWS Westwood ME Mallett S Deeks JJ Reitsma JB. QUADAS-2: A revised tool for the quality assessment of diagnostic accuracy studies. Ann Intern Med (2011) 155(8):529–36. doi: 10.7326/0003-4819-155-8-201110180-00009 22007046

[27] Bidgoli A DePriest BP Saatloo MV Jiang H Fu D Paczesny S. Current definitions and clinical implications of biomarkers in graft-versus-Host disease. Transplant Cell Ther (2022) 28(10):657–66. doi: 10.1016/j.jtct.2022.07.008 PMC9547856 35830932

[28] Gavriilaki E Chrysanthopoulou A Sakellari I Batsis I Mallouri D Touloumenidou T. Linking complement activation, coagulation, and neutrophils in transplant-associated thrombotic microangiopathy. Thromb Haemost. (2019) 119(9):1433–40. doi: 10.1055/s-0039-1692721 31266080

[29] Xu Z Luo C Lai P Ling W Wu S Huang X. Von willebrand factor as a predictor for transplant-associated thrombotic microangiopathy. Clin Appl Thromb Hemost. (2020) 26. doi: 10.1177/1076029619892684 PMC7256332 32088973

[30] Okamura H Nakamae H Shindo T Ohtani K Hidaka Y Ohtsuka Y. Early elevation of complement factor ba is a predictive biomarker for transplant-associated thrombotic microangiopathy. Front Immunol (2021) 12:695037. doi: 10.3389/fimmu.2021.695037 34326846 PMC8315095

[31] Horváth O Kállay K Csuka D Mező B Sinkovits G Kassa C. Early increase in complement terminal pathway activation marker sC5b-9 is predictive for the development of thrombotic microangiopathy after stem cell transplantation. Biol Blood Marrow Transplant. (2018) 24(5):989–96. doi: 10.1016/j.bbmt.2018.01.009 29339271

[32] Mezö B Horváth O Sinkovits G Veszeli N Kriván G Prohászka Z. Validation of early increase in complement activation marker sC5b-9 as a predictive biomarker for the development of thrombotic microangiopathy after stem cell transplantation. Front Med (2020) 7:646. 10.3389/fmed.2020.569291 PMC7574906 33117830

[33] Sartain S Shubert S Wu M-F Wang T Martinez C. The alternative complement pathway activation product ba as a marker for transplant-associated thrombotic microangiopathy. Pediatr Blood Cancer. (2020) 67(3):e28070. doi: 10.1002/pbc.28070 31774252

[34] Wall SA Zhao Q Yearsley M Blower L Agyeman A Ranganathan P. Complement-mediated thrombotic microangiopathy as a link between endothelial damage and steroid-refractory GVHD. Blood Adv (2018) 2(20):2619–28. doi: 10.1182/bloodadvances.2018020321 PMC6199668 30327370

[35] Qi J Wang J Chen J Su J Tang Y Wu X. Plasma levels of complement activation fragments C3b and sC5b-9 significantly increased in patients with thrombotic microangiopathy after allogeneic stem cell transplantation. Ann Hematol (2017) 96(11):1849–55. doi: 10.1007/s00277-017-3092-9 PMC6225065 28801815

[36] Li A Bhatraju PK Chen J Chung DW Hilton T Houck K. Prognostic biomarkers for thrombotic microangiopathy after acute graft-versus-Host disease: A nested case-control study. Transplant Cell Ther (2021) 27(4):308.e1–308.e8. doi: 10.1016/j.jtct.2020.12.010 PMC10122917 33836868

[37] Zeisbrich M Becker N Benner A Radujkovic A Schmitt K Beimler J. Transplant-associated thrombotic microangiopathy is an endothelial complication associated with refractoriness of acute GvHD. Bone Marrow Transplant. (2017) 52:1399. doi: 10.1038/bmt.2017.119 28650448

[38] Matsuda Y Hara J Osugi Y Tokimasa S Fujisaki H Takai K. Serum levels of soluble adhesion molecules in stem cell transplantation-related complications. Bone Marrow Transplant. (2001) 27(9):977–82. doi: 10.1038/sj.bmt.1703026 11436109

[39] Zeigler ZR Rosenfeld CS Andrews DF III Nemunaitis J Raymond JM Shadduck RK. Plasma von willebrand factor antigen (vWF:AG) and thrombomodulin (TM) levels in adult thrombotic thrombocytopenic purpura/hemolytic uremic syndromes (TTP/HUS) and bone marrow transplant-associated thrombotic microangiopathy (BMT-TM). Am J Hematol (1996) 53(4):213–20. doi: 10.1002/(SICI)1096-8652(199612)53:4<213::AID-AJH1>3.0.CO;2-0 8948657

[40] Arai Y Yamashita K Mizugishi K Watanabe T Sakamoto S Kitano T. Serum neutrophil extracellular trap levels predict thrombotic microangiopathy after allogeneic stem cell transplantation. Biol Blood Marrow Transplant. (2013) 19(12):1683–9. doi: 10.1016/j.bbmt.2013.09.005 24055655

[41] Pan T Qi J You T Han S Yang L Miao W. Circulating heme oxygenase-1 and complement activation in transplant-associated thrombotic microangiopathy. Biol Blood Marrow Transplant. (2019) 25(8):1486–91. doi: 10.1016/j.bbmt.2019.03.002 30871975

[42] Luft T Dreger P Radujkovic A. Endothelial cell dysfunction: a key determinant for the outcome of allogeneic stem cell transplantation. Bone Marrow Transplant. (2021) 56(10):2326–35. doi: 10.1038/s41409-021-01390-y PMC8273852 34253879

[43] Fosse JH Haraldsen G Falk K Edelmann R. Endothelial cells in emerging viral infections. Front Cardiovasc Med (2021) 8:619690. doi: 10.3389/fcvm.2021.619690 33718448 PMC7943456

[44] Rotz SJ Dandoy CE Davies SM. ST2 and endothelial injury as a link between GVHD and microangiopathy. N Engl J Med (2017) 376(12):1189–90. doi: 10.1056/NEJMc1700185 28328331

[45] Davies SM Gloude NJ Jodele S Khandelwal P Alder MN Lake KE. Circulating dsDNA, endothelial injury, and complement activation in thrombotic microangiopathy and GVHD. Blood. (2017) 130(10):1259–66. doi: 10.1182/blood-2017-05-782870 PMC5714230 28705839

[46] Betzmann D Doring M Blumenstock G Erdmann F Grabow D Lang P. Impact of pre-transplant serum-IGF-1 on hematopoietic stem cell transplantation outcome in pediatric cancer patients. Horm Res Paediatr (2021) 94(SUPPL 1):59. 10.1016/j.jtct.2022.03.027 35405367

[47] Luebbering N Abdullah S Lounder D Lane A Dole N Rubinstein J. Endothelial injury, f-actin and vitamin-d binding protein after hematopoietic stem cell transplant and association with clinical outcomes. Haematologica. (2021) 106(5):1321–9. doi: 10.3324/haematol.2019.233478 PMC8094097 32241849

[48] Postalcioglu M Kim HT Obut F Yilmam OA Yang J Byun BC. Impact of thrombotic microangiopathy on renal outcomes and survival after hematopoietic stem cell transplantation. Biol Blood Marrow Transplant. (2018) 24(11):2344–53. doi: 10.1016/j.bbmt.2018.05.010 PMC6230502 29758394

[49] Schuh MP Bennett MR Lane A Jodele S Laskin BL Devarajan P. Haptoglobin degradation product as a novel serum biomarker for hematopoietic stem cell transplant-associated thrombotic microangiopathy. Pediatr Nephrol. (2019) 34(5):865–71. doi: 10.1007/s00467-018-4178-x PMC6728916 30569313

[50] Zeigler ZR Kelton JG Moore JC Shadduck RK Andrews DF Nath R. Calpain activity in bone marrow transplant-associated thrombotic thrombocytopenic purpura. Bone Marrow Transplant. (1999) 24(6):641–5. doi: 10.1038/sj.bmt.1701928 10490730

[51] Jodele S Dandoy CE Sabulski A Koo J Lane A Myers KC. TA-TMA risk stratification: is there a window of opportunity to improve outcomes? Transplant Cell Ther (2022). doi: 10.1016/j.jtct.2022.04.019 PMC9351710 35490975

[52] Zhang X Liu X Wang Q He Y Zhu X Zhang J. Thrombotic microangiopathy with concomitant GI aGVHD after allogeneic hematopoietic stem cell transplantation: Risk factors and outcome. Eur J Haematol (2018) 100(2):171–81. doi: 10.1111/ejh.12996 29114931

[53] Uderzo C Fumagalli M De Lorenzo P Busca A Vassallo E Bonanomi S. Impact of thrombotic thrombocytopenic purpura on leukemic children undergoing bone marrow transplantation. Bone Marrow Transplant. (2000) 26:1005. doi: 10.1038/sj.bmt.1702648 11100281

[54] Zhao P Wu Y He Y Chong S Qu Q Deng R. A prognostic model (BATAP) with external validation for patients with transplant-associated thrombotic microangiopathy. Blood Adv (2021) 5(24):5479–89. doi: 10.1182/bloodadvances.2021004530 PMC8714708 34507352

[55] Gavriilaki E Sakellari I Chatzikonstantinou T Mallouri D Batsis I Vardi A. Endothelial and complement activation as predictors of survival in adult allogeneic hematopoietic cell transplantation. HemaSphere. (2021) 5(1). 10.1097/HS9.0000000000000487 PMC7732269 33324949

[56] Jekarl DW Kim Y Lim J Kim M Han K Cho B. Fragmented red cell as a possible favorable prognostic marker of hematopoietic stem cell transplantation associated thrombotic microangiopathy. J Clin Lab Anal (2015) 29(6):444–50. doi: 10.1002/jcla.21792 PMC6807144 25385174

[57] Cordes S Mokhtari Z Bartosova M Mertlitz S Shi Y Mengwasser J. Endothelial damage and dysfunction in acute graft-versus-host disease. Haematologica. (2021) 106(8):2147–60. 10.3324/haematol.2020.253716 PMC8327719 32675225

[58] Martinez-Sanchez J Hamelmann H Palomo M Mir E Moreno-Castaño AB Torramade S. Acute graft-vs.-Host disease-associated endothelial activation *in vitro* is prevented by defibrotide. Front Immunol (2019) 10:2339. doi: 10.3389/fimmu.2019.02339 31649666 PMC6794443

[59] Schisterman EF Cole SR Platt RW. Overadjustment bias and unnecessary adjustment in epidemiologic studies. Epidemiology. (2009) 20(4):488–95. doi: 10.1097/EDE.0b013e3181a819a1 PMC2744485 19525685

[60] Ardissino G Capone V Tedeschi S Porcaro L Cugno M. Complement system as a new target for hematopoietic stem cell transplantation-related thrombotic microangiopathy. Pharmaceuticals. (2022) 15(7). doi: 10.3390/ph15070845 PMC9325021 35890144

[61] Jodele S. Complement in pathophysiology and treatment of transplant-associated thrombotic microangiopathies. Semin Hematol (2018) 55(3):159–66. doi: 10.1053/j.seminhematol.2018.04.003 30032753

