# Supplementary material for: A systematic review of diagnostic, prognostic, and risk blood and urine biomarkers of transplant-associated thrombotic microangiopathy
Source: Front Immunol. 2023 Feb 2;13:1064203. doi: 10.3389/fimmu.2022.1064203 (PMC9933706; doi:10.3389/fimmu.2022.1064203)
Supplement: Supplementary file 1 [file Table_1.docx]

**Supplement Table 1: Search Strategy**

| thrombotic AND microangiopathy AND bone AND marrow AND transplant |
| --- |
| 'biomarker ttp bone marrow transplant' OR (('biomarker'/exp OR biomarker OR thrombotic) AND thrombocytopenia AND purpura AND ('bone'/exp OR bone) AND ('marrow'/exp OR marrow) AND ('transplant'/exp OR transplant)) |
| 'biomarker bone marrow transplant thrombotic microangiopathy' OR (('biomarker'/exp OR biomarker) AND ('bone'/exp OR bone) AND ('marrow'/exp OR marrow) AND ('transplant'/exp OR transplant) AND thrombotic AND ('microangiopathy'/exp OR microangiopathy)) |
| biomarker AND transplant AND associated AND thrombotic AND microangiopathy |
| 'biomarker bone marrow transplant thrombotic microangiopathy' OR (('biomarker'/exp OR biomarker) AND ('bone'/exp OR bone) AND ('marrow'/exp OR marrow) AND ('transplant'/exp OR transplant) AND thrombotic AND ('microangiopathy'/exp OR microangiopathy)) |
| 'biomarker bone marrow transplant thrombotic microangiopathy' OR (('biomarker'/exp OR biomarker) AND ('bone'/exp OR bone) AND ('marrow'/exp OR marrow) AND ('transplant'/exp OR transplant) AND thrombotic AND ('microangiopathy'/exp OR microangiopathy)) |
| 'biomarker thrombotic microangiopathy hematopoietic cellular therapy' OR (('biomarker'/exp OR biomarker) AND thrombotic AND ('microangiopathy'/exp OR microangiopathy) AND hematopoietic AND cellular AND ('therapy'/exp OR therapy)) |
| 'biomarker transplant associated thrombotic microangiopathy' OR (('biomarker'/exp OR biomarker) AND ('transplant'/exp OR transplant) AND associated AND thrombotic AND ('microangiopathy'/exp OR microangiopathy)) |

**Supplemental Table 2: References for biomarkers overlapping with TA-TMA biomarkers identified in this review**

| Biomarker | SOS | GVHD | Acute Respiratory Failure |
| --- | --- | --- | --- |
| sC5b-9 |  | ^27^ | ^53,54^ |
| C3b |  | ^34^ | ^54^ |
| mHam |  | ^27^ | ^55^ |
| CH50 |  |  | ^56^ |
| Ba |  |  | ^53,57^ |
| Bb |  |  | ^57^ |
| TM | ^58^ | ^59^ | ^53^ |
| sVCAM-1 | ^60^ | ^61^ | ^62^ |
| ANG-2 | ^60^ | ^59^ | ^53,63^ |
| ST2 | ^60^ | ^23,64,65^ | ^66,67^ |
| vWF %, vWF:ag | ^68^ | ^69^ | ^537071^ |
| LDH |  | ^72^ | ^73^ |
| Nitrates | ^74^ | ^75^ | ^76^ |
| Insulin-like growth factor-1 | ^77,78^ |  | ^79^ |
| Heme-Oxygenase-1 |  | ^80^ | ^81^ |
| dsDNA (NETS) |  |  | ^82,83^ |
| EASIX | ^84^ | ^84^ | ^85^ |

**Supplemental Table 3: Kits used to measure biomarkers**

| Reference | Study (year) | Biomarker Kits |
| --- | --- | --- |
| 2 | Jodele (2014) | ELISA – Cincinnati children’s lab |
| 9 | Vasu (2022) | ELISA kits |
| 14 | Uderzo (2006) | -- |
| 15 | Schoettler (2020) | Clinical Labs |
| 28 | Gavriilaki (2019) | ELISA kit (Quidel, San Diego, USA) |
|  |  | Human sVCAM-1/CD106 Quantikine ELISA Kit (R&D Systems) |
|  |  | iMark Microplate Absorbance Reader (Bio-Rad, Hercules, California, United States) |
|  |  | Human Thrombomodulin/BDCA-3 Quantikine ELISA Kit |
| 29 | Xu (2020) | Immune turbidimetric assay kit (STA Liatest, DIAGNOSTICA STAGO, Asnières-sur-Seine, France) on STA-R Evolution analyzer (DIAGNOSTICA STAGO, Asnières-sur-Seine, France) |
| 30 | Okamura (2021) | MicroVue Ba Enzyme Immunoassay Kit |
| 31 | Horvath (2018) | MicroVue sC5b-9 Plus EIA – Quidel |
| 33 | Sartain (2019) | ELISA (A033, Quidel, United States) |
| 34 | Wall (2018) | - |
| 35 | Qi (2017) | ELISA (Xitang, Shanghai, China) |
| 36 | Li (2020) | sC5b9 - MicroVue ELISA kit – Quidel |
|  |  | ANG-2 - Electro chemiluminescent immunoassays (Meso Scale Discovery, Rockville, MD) |
| 37 | Zeisbrich (2017) | ELISA kits (R&D Systems Europe Ltd, Abingdon, UK) |
| 38 | Matsuda (2001) | ELISA kit from R & D Systems (Minneapolis, MN, USA) |
| 40 | Arai (2013) | Capture ELISA [Quant-iT PicoGreen double-stranded DNA (dsDNA) Reagent (#P7581, Molecular Probes, Eugene, OR)] |
| 41 | Pan (2019) | Human HO-1 ELISA kits (Ameko, Hu, China) |
| 42 | Rotz (2017) | ELISA (Plasma R & D Systems ELISA in cohorts 1 and 3, serum Critical Diagnostics ELISA in cohort 2) |
| 43 | Gloude (2017) | Quant-iT PicoGreen dsDNA Reagent and Kit (Cat. no. P7589; Molecular Probes, Eugene, OR) |
| 46 | Uderzo (2000) | - |
| 48 | Jekarl (2015) | Sysmex XE 2100 hematologic analyzer (Sysmex, Kobe, Japan) |
| 56 | Mezo (2020) | ELISA kit |
| 57 | Zeigler (1996) | ELISA (Diagnostica Stago, Asnieres-sur-Seine, France) |
| 58 | Gavriilaki (2021) | Serum levels of LDH, Creatinine, and Platelets |
| 59 | Betzmann (2022) | Radioimmuno assay (in-house University Children's Hospital Tübingen, Tübingen, Germany) |
| 60 | Luebbering (2021) | ELISA (MyBioSource, 702018) |
| 61 | Postalcioglu (2018) | - |
| 62 | Schuh (2019) | Western Blot. Abcam polyclonal rabbit anti-haptoglobin antibody (1:2000) and Novex goat anti-rabbit HRP conjugate secondary antibody (1:50,000). Antibodies were diluted in SuperBlock (PBS) Blocking Buffer (Thermo Scientific, Waltham, MA) |
| 63 | Jodele (2022) | CCHMC Laboratory |
| 64 | Zeigler (1999) | Radiolabeling with ^14^C-serotonin 1.67 3 1024 mCi/106 platelets (Amersham, Oakville, Ontario, Canada). |
| 65 | Zhang (2018) | - |
| 66 | Zhao (2021) | - |
